# Case report: Single-operator peroral cholangioscopy system (SpyGlass) diagnosis of an extrahepatic biliary cystadenoma (video)

**DOI:** 10.3389/fmed.2023.1175034

**Published:** 2023-06-22

**Authors:** Xin Deng, Jingwen Wang, Tong Mou, Long Pan, Chengyou Du, Qiao Wu

**Affiliations:** ^1^Department of Hepatobiliary Surgery, The First Affiliated Hospital of Chongqing Medical University, Chongqing, China; ^2^Department of Neurology, The First Affiliated Hospital of Chongqing Medical University, Chongqing, China

**Keywords:** biliary cystadenoma, SpyGlass, ERCP, cholangioscopy, indeterminate strictures

## Abstract

Biliary cystadenoma is a type of rare liver cystic tumor. Intrahepatic biliary cystadenomas are the most common, while extrahepatic biliary cystadenomas are rarely seen. Biliary cystadenoma tends to occur in middle-aged to older women and there is a lack of specific preoperative diagnostic markers. Recent advancements in technology and the development of the SpyGlass system have led to an increased use of cholangioscopy. Herein, we report a patient in whom a space-occupying lesion was found in the bile duct by SpyGlass, and who later underwent radical surgery. The pathology report indicated that the final diagnosis was biliary cystadenoma. SpyGlass cholangioscopy may be a novel and effective diagnostic method for biliary cystadenoma.

## Introduction

Biliary cystadenoma, which is considered a rare disease, mostly occurring in the intrahepatic bile duct (80–85%), and rarely occurs in the extrahepatic bile duct (1, 2). The tumor is usually multilocular, and the inner layer of the cyst wall consists of columnar or cuboidal mucosal epithelial cells that can secrete copious amounts of mucus. The subepithelial layer consists of a dense stromal cell layer that can produce ovarian-like stroma. Biliary cystadenoma tends to occur in women around the age of 40 years; it is rare in children and adolescents, and may undergo malignant transformation (2, 3). Biliary cystadenoma is rarely seen in clinical practice and does not often have a specific clinical presentation. There is also a lack of specific laboratory test markers; hence there is a high tendency that biliary cystadenoma is misdiagnosed as other cystic tumors in preoperative imaging. Cholangioscopic technique has evolved over the last decade and since 2007, the first digital single-operator peroral cholangioscope (SpyGlass™ DS System, Boston Scientific, Marlborough, MA, U.S.) has been available offering biliary stone treatment using electrohydraulic lithotripsy or laser lithotripsy. In 2015, the second-generation SpyGlass was developed. It could further improve diagnostic capabilities upon the first-generation by providing four times higher image resolution and wider field-of-view (4, 5). Herein we report a case of biliary cystadenoma in the common bile duct discovered by endoscopic retrograde cholangiopancreatography (ERCP) combined with SpyGlass cholangioscopy.

## Case report

The patient was a 54-year-old female who was admitted for “jaundice for more than 10 days.” She was previously healthy and did not have a medical history of schistosomiasis.

**Figure 1:**
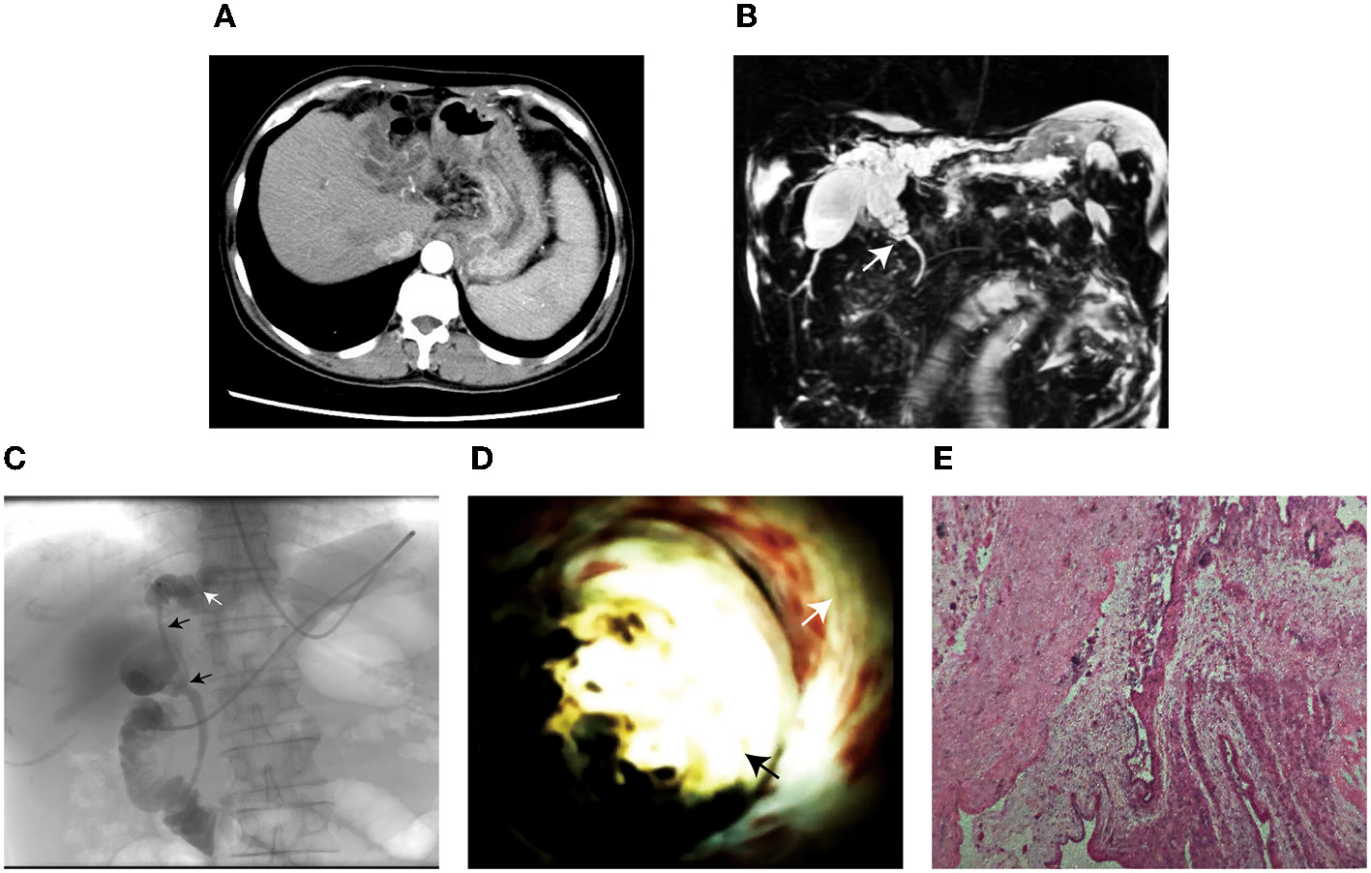
**(A)** Enhanced CT showed the dilation of intrahepatic bile duct. **(B)** MRCP showed the common bile duct stricture in the head of the pancreas (white arrow), additional soft tissue shadows were not seen at the surrounding regions and the upstream common bile duct and intrahepatic bile duct dilated significantly. **(C)** ERCP indicated that biliary stricture was at the upper common bile duct and hilar bile duct (black arrows) and left intrahepatic bile duct dilated significantly. **(D)** SpyGlass showed that an oval space-occupying lesion was seen at the upper segment of the common bile duct (black arrow). The biliary wall (white arrow) was smooth. The lesion filled the bile duct lumen, resulting in bile duct obstruction. The surface of this lesion was smooth, without dilated and tortuous blood vessels. **(E)** Postoperative pathological examination revealed secretory biliary cystadenoma.

Following were results of the laboratory tests: liver function tests were abnormal, with alanine aminotransferase levels of 71 U/L (normal range: 14–54 U/L), aspartate aminotransferase levels of 104 U/L (normal range: 15–41 U/L), and a total bilirubin of 72 μmol/L (normal range: 0–16 μmol/L), HbsAg (+), HbeAg (+), HbsAb (+). Carbohydrate antigen 19-9 (CA19-9) was moderately elevated (103 U/ml); and other tumor markers were normal. Ultrasound showed the dilation of extrahepatic and intrahepatic bile ducts caused by abnormal choledochal echo. Moreover, the computed tomography (CT) (Figure 1A) and magnetic resonance cholangiopancreatography (MRCP) (Figure 1B) demonstrated that the common bile duct stenosis located at the head of the pancreas and the upstream bile duct dilatation was significant. As the patient's diagnosis was unclear, she underwent a ERCP with adequate preoperative preparations. The biliary cholangiography revealed an indeterminate stenosis at the upper bile duct (Figure 1C). Further evaluation of SpyGlass cholangioscopy (Boston Scientific, Marlborough, MA, U.S.) showed an oval space-occupying lesion at the upper segment of the common bile duct. The surface mucosa of the lesion was smooth, without neovascularization (Figure 1D and Supplementary Video S1). Four days after ERCP and endoscopic nasobiliary drainage (ENBD), the patient underwent radical surgery. A palpable hard mass was found at the hepatic hilum along the common bile duct during surgery. The tumor invaded the confluence of the left and right bile duct. Samples were dissected postoperatively and a 5 × 3 × 2 cm mass was found in the common bile duct and the upper end of the mass invaded the start of the left hepatic duct. Postoperative pathology examination revealed secretory cystadenoma in the common bile duct. Immunohistochemistry results showed a CD10 (+), ER (+), PR weak (–), and Ki67: 5% (+) secretory cystadenoma (Figure 1E). After more than two-year follow-up postresection, the patient has been doing well, without any evidence of recurrence.

## Discussion

Biliary cystadenoma is a type of rare benign tumor with a high recurrence rate and potential for malignant transformation. Disease onset is slow and there are no typical clinical symptoms. This disease is considered to have the potential for malignant transformation and is mostly found in middle-aged women. The proportion of female patients is over 90% and most tumors are located in the intrahepatic bile duct (3). The world's first case of this disease was reported by Hueter in 1887. Five years later, Keen reported the world's first case of biliary cystadenoma resected surgically (6). At present, very few cases have been reported. It is believed that biliary cystadenoma is associated with congenital biliary developmental abnormalities and ectopic ovarian stroma. Some researchers believed that biliary cystadenoma is associated with oral contraceptives. However, some studies found that most patients did not have a history of oral contraceptive use (7, 8). The clinical manifestations of biliary cystadenoma are mainly determined by the size and site of the tumor and are mostly non-specific, including abdominal pain, abdominal distension, and discomfort in the liver area. It should be pointed out that the most common clinical manifestations of extrahepatic biliary cystadenoma are abdominal pain and jaundice (1, 9, 10). The routine examinations for biliary cystadenoma include ultrasound, CT and magnetic resonance imaging (MRI). Preoperative radiologic examination results are the main basis for preoperative diagnosis. Most ultrasound examination results of biliary cystadenoma show cystic structures with clear borders and heterogeneous echoes that are segmented, which can be seen in the mass (11). Non-enhanced CT scan will show a hypodense lesion with clear borders and multiple septa, mild enhancement at the arterial phase cystic walls and septa, and continuous enhancement during the venous phase (12). MRI T1-weighted imaging shows cystic hypodense signals with segmentation inside the mass. T2-weighted imaging shows high intensity signals and low intensity septa, mild enhancement in the arterial phase, and continuous enhancement in the venous phase (13). Laboratory tests may show abnormal hepatic function, elevated alanine aminotransferase, aspartate aminotransferase, alkaline phosphatase, and gamma glutamyl transferase. The tumor markers CA19-9 and CEA may be elevated; however, these have no diagnostic value. Due to a lack of a typical clinical presentation, specific laboratory tests, and radiologic examination methods, the final diagnosis of biliary cystadenoma is still dependent on pathological examinations. A previous study showed that biliary cystadenoma originates from embryonic precursor cells at the biliary epithelium and 90% of biliary cystadenoma are located in the intrahepatic bile ducts, while very few cases occur at the extrahepatic bile ducts (14). Biliary cystadenomas are mostly polyp-like protrusions located at the biliary walls or are unilocular or multilocular protrusions filled with mucus or serous fluid. Under microscopy, a monolayer of cuboidal cells could be seen at the tumor surface and their cell nuclei tend to be located at the base of the cell. Intrahepatic biliary cystadenoma mostly presents as large multilocular or unilocular space-occupying lesions, while extrahepatic biliary cystadenoma mostly presents as multilocular or unilocular space-occupying lesions or small vesicles at the biliary wall. Radical resection is used as the mainstay treatment for biliary cystadenoma, regardless of whether it is benign or malignant.

In the present case, obstructive jaundice was the main presentation. The site of obstruction was the upper and middle segments of the common bile duct. Both magnetic resonance cholangiopancreatography and enhanced CT indicated common bile duct stenosis accompanied by mild CA19-9 elevation. Timely and accurate qualitative diagnosis is critical and determines the treatment protocol. Biliary imaging alone is usually insufficient for a definitive diagnosis and transoral cholangioscopy is necessary for the direct and accurate examination. Since the emergence of the SpyGlass, the sensitivity for detecting cholangiocarcinoma has increased, thereby establishing its superiority over standard ERCP. ERCP-guided cholangiopancreatoscopy with SpyGlass allows for direct visualization of the bile ducts, tissue sampling and therapeutic maneuvers (15, 16). Previous studies have shown that the diagnostic accuracy of spyglass visual impression could reach 80.0–89.0% (15, 17, 18). One meta-analysis reported that the sensitivity and specificity of SpyGlass in diagnosing indeterminate biliary strictures were 94 and 95%, respectively (19). This present case highlights the importance of SpyGlass in the diagnosis of indeterminate biliary lesions.

In summary, biliary cystadenomas, particularly extrahepatic biliary cystadenomas, have a low incidence, do not have a typical clinical presentation, and misdiagnosis and missed diagnosis are common. Clinicians should therefore improve their understanding of this disease. With widespread clinical use of SpyGlass, it can greatly improve the diagnostic accuracy of biliary diseases, particularly rare diseases. Meanwhile, early diagnosis and proper treatment can improve patient prognosis.

## Data availability statement

The original contributions presented in the study are included in the article/Supplementary material, further inquiries can be directed to the corresponding author.

## Ethics statement

Ethical review and approval was not required for the study on human participants in accordance with the local legislation and institutional requirements. The patients/participants provided their written informed consent to participate in this study. Written informed consent was obtained from the individual(s) for the publication of any potentially identifiable images or data included in this article.

## Author contributions

All authors listed have made a substantial, direct, and intellectual contribution to the work and approved it for publication.

## References

[1] Delis SG Touloumis Z Bakoyiannis A Tassopoulos N Paraskeva K Athanassiou K. Intrahepatic biliary cystadenoma: a need for radical resection. Eur J Gastroenterol Hepatol. (2008) 20:10–4. 10.1097/MEG.0b013e3282f16a76 18090983

[2] Devaney K Goodman ZD Ishak KG. Hepatobiliary cystadenoma and cystadenocarcinoma. A light microscopic and immunohistochemical study of 70 patients. Am J Surg Pathol. (1994) 18:1078–91. 10.1097/00000478-199411000-00002 7943529

[3] Voltaggio L Szeto OJ Tabbara SO. Cytologic diagnosis of hepatobiliary cystadenoma with mesenchymal stroma during intraoperative consultation: a case report. Acta Cytol. (2010) 54:928–32. 21053571

[4] Bokemeyer A Gerges C Lang D Bettenworth D Kabar I Schmidt H. Digital single-operator video cholangioscopy in treating refractory biliary stones: a multicenter observational study. Surg Endosc. (2020) 34:1914–22. 10.1007/s00464-019-06962-0 31309312

[5] Yodice M Choma J Tadros M. The expansion of cholangioscopy: established and investigational uses of SpyGlass in biliary and pancreatic disorders. Diagnostics. (2020) 10:132. 10.3390/diagnostics10030132 32121412 PMC7151166

[6] Henson SW Jr Gray HK Dockerty MB. Benign tumors of the liver VI Multilocular cystadenomas. Surg Gynecol Obstet. (1957) 104:551–4. 13433250

[7] Lee JH Lee KG Park HK Lee KS. Biliary cystadenoma and cystadenocarcinoma of the liver: 10 cases of a single center experience. Hepatogastroenterology. (2009) 56:844–9. 19621714

[8] Emre A Serin KR Ozden I Tekant Y Bilge O Alper A. Intrahepatic biliary cystic neoplasms: surgical results of 9 patients and literature review. World J Gastroenterol. (2011) 17:361–5. 10.3748/wjg.v17.i3.361 21253396 PMC3022297

[9] Kim HH Hur YH Koh YS Cho CK Kim JW. Intrahepatic biliary cystadenoma: Is there really an almost exclusively female predominance? World J Gastroenterol. (2011) 17:3073–4. 10.3748/wjg.v17.i25.3073 21799657 PMC3132262

[10] Diaz de Liano A Olivera E Artieda C Yarnoz C Ortiz H. Intrahepatic mucinous biliary cystadenoma. Clin Transl Oncol. (2007) 9:678–80. 10.1007/s12094-007-0122-4 17974530

[11] Xu HX Lu MD Liu LN Zhang YF Guo LH Liu C. Imaging features of intrahepatic biliary cystadenoma and cystadenocarcinoma on B-mode and contrast-enhanced ultrasound. Ultraschall Med. (2012) 33:E241–E9. 10.1055/s-0031-1299276 23154870

[12] Kim JY Kim SH Eun HW Lee MW Lee JY Han JK. Differentiation between biliary cystic neoplasms and simple cysts of the liver: accuracy of CT. AJR Am J Roentgenol. (2010) 195:1142–8. 10.2214/AJR.09.4026 20966320

[13] Cogley JR Miller FH MR. imaging of benign focal liver lesions. Radiol Clin North Am. (2014) 52:657–82. 10.1016/j.rcl.2014.02.005 24889166

[14] Metussin A Telisinghe P Kok K Chong V. Extrahepatic biliary cystadenoma: a rare cause of biliary obstruction. Oman Med J. (2015) 30:66–8. 10.5001/omj.2015.13 25830005 PMC4371457

[15] Chen YK Parsi MA Binmoeller KF Hawes RH Pleskow DK Slivka A. Single-operator cholangioscopy in patients requiring evaluation of bile duct disease or therapy of biliary stones (with videos). Gastrointest Endosc. (2011) 74:805–14. 10.1016/j.gie.2011.04.016 21762903

[16] Theodoropoulou A Vardas E Voudoukis E Tavernaraki A Tribonias G Konstantinidis K. SpyGlass direct visualization system facilitated management of iatrogenic biliary stricture: a novel approach in difficult cannulation. Endoscopy. (2012) 44(Suppl. 2):E433–4. 10.1055/s-0032-1325857 23258490

[17] Chen YK Pleskow DK. SpyGlass single-operator peroral cholangiopancreatoscopy system for the diagnosis and therapy of bile-duct disorders: a clinical feasibility study (with video). Gastrointest Endosc. (2007) 65:832–41. 10.1016/j.gie.2007.01.025 17466202

[18] Ramchandani M Reddy DN Gupta R Lakhtakia S Tandan M Darisetty S. Role of single-operator peroral cholangioscopy in the diagnosis of indeterminate biliary lesions: a single-center, prospective study. Gastrointest Endosc. (2011) 74:511–9. 10.1016/j.gie.2011.04.034 21737076

[19] de Oliveira P de Moura DTH Ribeiro IB Bazarbashi AN Franzini TAP Dos Santos MEL. Efficacy of digital single-operator cholangioscopy in the visual interpretation of indeterminate biliary strictures: a systematic review and meta-analysis. Surg Endosc. (2020) 34:3321–9. 10.1007/s00464-020-07583-8 32342216

